# Mitigation of non-communicable diseases in developing countries with community health workers

**DOI:** 10.1186/s12992-015-0129-5

**Published:** 2015-11-10

**Authors:** Shiva Raj Mishra, Dinesh Neupane, David Preen, Per Kallestrup, Henry B. Perry

**Affiliations:** Nepal Development Society (NEDS), Bharatpur-10, Chitwan, Nepal; School of Population Health, The University Western Australia, 35 Stirling Hwy, Crawley, WA 6009 Australia; Department of Public Health, Center for Global Health, Aarhus University, Aarhus, Denmark; Department of International Health, Health Systems Program, Johns Hopkins Bloomberg School of Public Health, Baltimore, MS USA

**Keywords:** Community health workers, Lay health workers, Health system, Non-communicable diseases, Developing countries

## Abstract

Non-communicable diseases (NCDs) are rapidly becoming priorities in developing countries. While developed countries are more prepared in terms of skilled human resources for NCD management, developing the required human resources is still a challenge in developing countries. In this context, mobilizing community health workers (CHWs) for control of NCDs seems promising. With proper training, supervision and logistical support, CHWs can participate in the detection and treatment of hypertension, diabetes, and other priority chronic diseases. Furthermore, advice and support that CHWs can provide about diet, physical activity, and other healthy lifestyle habits (such as avoidance of smoking and excessive alcohol intake) have the potential for contributing importantly to NCD programs. This paper explores the possibility of involving CHWs in developing countries for addressing NCDs.

## Background

The global burden of non-communicable diseases (NCDs) poses major challenges for health systems in low and middle income countries (LMIC) [1]. Macroeconomic simulations suggest a cumulative output loss of US$ 47 trillion from the burden of NCDs over the next two decades, which is equal to 75 % of global GDP in 2010 (US$ 63 trillion) [2]. NCDs were estimated to have caused 68 % of the world’s 56 million deaths in 2012 and of these 28 million occurred in LMICs [3].

Despite being major causes of mortality and morbidity in many developing countries, NCDs have not yet become a priority for prevention, control and treatment in many of these nations [4]. NCDs largely have their roots in unhealthy lifestyles and adverse physical and social environments. Well-known risk factors include poor diets, smoking, physical inactivity, excessive use of alcohol, psychological stress, excessive consumption of calories, fats, and high-cholesterol containing foods [5]. Studies have demonstrated that the burden of NCDs can be reduced through effective preventive measures -- up to 30 % for cancer and 75 % for cardiovascular diseases [6]. For example, in countries such as China over 50 % of the increased NCD burden could be preventable by modifying behavioral risks [7]. There is well established knowledge on what should be done for the prevention of these diseases [8] . However, key questions still remain relating to how it should be done and how our existing knowledge of NCDs can best be applied for effective prevention in real-life situations, particularly in low-income countries. Here, we propose that community health workers (CHWs) can play an important role in addressing NCDs.

### Involving CHWs for addressing NCDs in developing countries

According to the recent estimates, there are five million CHWs worldwide, including 2.3 million in India alone [9]. A campaign conducted jointly by United Nations agencies, civil societies, the private sector, and academia is currently underway to train one million CHWs in Africa [10]. They are a diverse category of health workers who commonly work in communities outside of established health facilities and have some type of formal, but limited, training for the tasks they are expected to perform. The most complete description available of CHW programs in low-income countries has recently been released for Afghanistan (CHWs), Bangladesh (Shasthya Shebikas, Family Welfare Assistants, Health Assistants, and Community Health Care Providers), Brazil (Community Health Agents), Ethiopia (Health Extension Workers and Health Development Army Volunteers), India (Auxiliary Nurse Midwives, Anganwadi Workers, Accredited Social Health Activists, Multipurpose Workers, and Lady Health Visitors), Indonesia (Kaders), Iran (Behvarzs), Nepal (Village Health Workers, Maternal and Child Health Workers, and Female Community Health Volunteers), Pakistan (Lady Health Workers), Rwanda (Binômes/CHWs), Zambia (Health Assistants), and Zimbabwe (Village Health Workers) [11]. Given the existing shortage of higher-level health professionals in developing countries [12] and the difficulty of their deployment in rural settings, CHWs comprise a possible useful health resource for the long-term and are a rapidly growing component of the health workforce in many countries [13].

Expanding the capabilities of health systems to respond to NCDs is a challenge, especially in low-income countries. There has been an increasing focus on task shifting for maternal, child health and HIV services [14, 15]. However, there is still a dearth of information available on task shifting specifically in managing NCDs in low- to LMICs. A detailed systematic review by Joshi et al. concluded that when accompanied by health system re-structuring, task shifting is a potentially effective and affordable strategy for improving access to healthcare for NCDs [16]. The World Health Organization (WHO) recommendations on using less skilled workers to address the burden of HIV came as an effort to devolve the role of physician centric care to community health workers, also highly relevant today for other services including NCD [17]. For mental health conditions, the WHO has proposed the Mental Health Gap Action Programme (mhGAP) to translate the available evidence into simple clinical protocols for decision making and clinical assessments for health-care providers working in non-specialized health-care settings [18].

CHW intervention can address broader social determinants of health including behavioral factors. Trained CHWs have been effective for hypertension and diabetes control in Iran [19]. In Pakistan, CHW-delivered health promotion significantly reduced the onset of hypertension in children and young adults [20]. Earlier studies have shown that community-based approaches provide significant impact, not only because they are cost effective [21, 22], but particularly because these interventions establish community ownership and are sustainable [23]. While some countries have already commenced efforts to address the NCD challenge by implementing taxation laws on tobacco and alcohol, and offering facilities for physical activity [24], one of the most potentially cost effective approaches – engaging communities and CHWs in the response to NCDs – has not yet been prioritized.

CHWs can provide a strong link between the health system and communities. Many communities in developing countries are geographically dispersed with poor access to health services, have low literacy levels, and commonly do not receive information about the prevention, control and treatment of diseases, including NCDs. Specialist health workers working among people from a culture which is markedly different from their own often have to adapt somewhat to the local community’s language and customs, which could be challenging for many. In such situations, health professionals, even if they were available, may not succeed in adopting a culturally acceptable approach to address NCDs. In addition, lack of infrastructure, higher expectation for salary, lack of career enhancement opportunities are some of the reasons why specialist workers may prefer not to work in rural and developing setting [25, 26].

Lessons for treatment of NCDs can be learned from the deployment of CHWs over the past several decades that has led to significant reductions in maternal and child deaths in countries of Asia and Africa [9, 27, 28]. CHWs help make basic health services more available and affordable [22, 29, 30], thereby firmly supporting the principle of primary health care that calls for services to be provided in a way that is easily accessible, culturally appropriate, and sustainable [31]. Advice that CHWs can provide about diet, physical activity, and other healthy lifestyle habits in addition to the importance of proactive health service engagement for the ongoing management of chronic diseases has the potential to contribute importantly to NCD reduction and management programs. Also, if appropriately trained, CHWs can be instrumental in providing counseling for psychological problems and alcohol dependence. Peer-to-peer health promotion and engagement of participatory women’s groups led by CHWs have proven highly successful for improving maternal and child health [32–34]. Moreover, CHWs have advantages in terms of accessibility to patients as they can provide services at both established health posts and through direct home visitation.

With the appropriate training, supervision and logistical support, CHWs could carry out a range of activities including : i) health promotion for reduction of risk factors for NCDs; ii) screening of households and special targeted health promotion for hypertension, diabetes (or a high-risk of diabetes based on age, family history, weight, and level of physical activity) [35]) and serious mental illness (including depression); iii) treatment using a simple clinical algorithm, with appropriate supervision and support. They could also be trained to measure blood pressure and blood glucose if provided with the required measuring equipment and foot check for diabetic ulcers [16]. Performing all these activities adds little more to the total costs in health systems. An economic evaluation of task shifting for treatment of depressive disorders by lay health workers in India found that use of lay health workers were not just cost effective but cost saving as well [36]. There are challenges in CHW mobilization for NCD control. First of all, overloading of CHWs with additional tasks might not just be detrimental to the quality of work they perform, but their physical and emotional health. Increasing the number of routine services above those they are already providing presents the risk of resulting in less time devoted to essential services and the quality of the service may ultimately be reduced. Adding more tasks to the CHW portfolio, such as chronic disease identification and management, could further aggravate these concerns. However, CHW programs need to be able to adapt over time in accordance with the changing health needs of the population, including adding more CHWs as the workload increases. This could include giving CHWs more specialized roles at the community level, as is already being done in some countries such as Rwanda where female CHWs focus more on reproductive, maternal and child health issues [37]. All of these issues can be managed adequately if the CHW program is adequately resourced, if the program responds to the felt concerns of the CHWs, and if the program adjusts to address local health priorities.

Another challenge concerns ethical issues involved with transferring the responsibility of providing health care to CHWs who do not receive as much training as auxiliaries, nurses or physicians. Involving CHWs as health care providers might reduce the quality of health services. However, government regulation of CHW activities can ensure that CHWs who are diagnosing and treating sick patients or who are providing services with potential serious side effects are appropriately trained and supervised. CHWs should not be performing certain tasks as independent practitioners without clinical oversight and regular quality assessment. This is to say that governments, with the input of clinical regulatory and health professional bodies, need to establish national guidelines and regulations under which CHW programs operate, whether they are government programs or private programs. Experienced CHWs themselves should participate in developing these regulations and standards. A taskforce on management and continuous improvement of CHW programs can collaborate in establishing regulations as well as designing, implementing and monitoring programs. The process of stronger regulation is now occurring in many countries in Sub-Saharan Africa [38].

When expanding the number of tasks that CHWs perform, a dilemma exists whether to recruit new cadres of CHWs or to reorient the currently functioning CHWs that many countries have had for some time and perhaps increase their number. In many cases, CHWs would be offering a service that was previously unavailable. Since community-based NCD programs are still largely in their infancy in many LMICs, it is too early to know whether it is preferable to have new CHWs who specialize only in NCDs or to add NCD activities to the list of duties of currently functioning CHWs. More community-based interventional studies are needed to explore the effectiveness of CHW programs as they take on expanded responsibilities for NCD detection, prevention and treatment. However, it has been suggested that over the next few decades, the burden of work related to maternal and child health and to infectious diseases will decline in most LMICs [39], allowing CHWs to focus on other priorities. However, the health burdens from NCDs and health issues of the elderly will also grow, so it is critical that CHWs have the capacity to respond to these new health challenges.

## Conclusion

As a result of the vast growing network of CHWs in developing countries, the potential of a community response to NCDs seems promising [7]. CHWs have a respected position in their local communities and can be effectively integrated into existing health systems. In order to be effective, CHWs must be well-trained, well-supervised, and given the supplies and medicines they need to perform their tasks [11]. Several challenges remain: (1) to not over burden CHWs, (2) not to diminish the quality of care of the services they currently provide as they take on new tasks, and (3) determine whether to recruit an entirely new cadre of CHWs for NCD management or work with existing cadres. Empirical studies are needed to test the effectiveness of CHWs in NCD prevention, detection and management [40]. CHW programs, if they are going to be effective, must be adequately resourced and have strong monitoring, evaluation and quality-assurance systems so that they can continually improve. CHW programs are not a cheap option, but they are comparatively less expensive than the alternative option, which is to provide these services at readily available facilities by higher-level staff. Not only is this latter option more expensive, it will also take much more time to implement fully and available workforce issues would be a limiting factor. Moreover, vigorous discussions are necessary at all levels to develop community-oriented NCD policies, strategies and programs in developing countries that make full use of the potential that CHWs hold.
